# Antifungal Efficacy of Redox-Active Natamycin against Some Foodborne Fungi—Comparison with *Aspergillus fumigatus*

**DOI:** 10.3390/foods10092073

**Published:** 2021-09-02

**Authors:** Jong H. Kim, Christina C. Tam, Kathleen L. Chan, Luisa W. Cheng, Kirkwood M. Land, Mendel Friedman, Perng-Kuang Chang

**Affiliations:** 1 Foodborne Toxin Detection and Prevention Research Unit, Western Regional Research Center, USDA-ARS, 800 Buchanan St., Albany, CA 94710, USA; christina.tam@usda.gov (C.C.T.); kathy.chan@usda.gov (K.L.C.); luisa.cheng@usda.gov (L.W.C.); 2 Department of Biological Sciences, University of the Pacific, 3601 Pacific Avenue, Stockton, CA 95211, USA; kland@pacific.edu; 3 Healthy Processed Foods Research Unit, Western Regional Research Center, USDA-ARS, 800 Buchanan St., Albany, CA 94710, USA; mendel.friedman@usda.gov; 4 Food and Feed Safety Research Unit, Southern Regional Research Center, USDA-ARS, 1100 Robert E. Lee Boulevard, New Orleans, LA 70124, USA; perngkuang.chang@usda.gov

**Keywords:** antifungal, natamycin, oxidative stress, pH, polyenes, redox-active, drug resistance

## Abstract

The fungal antioxidant system is one of the targets of the redox-active polyene antifungal drugs, including amphotericin B (AMB), nystatin (NYS), and natamycin (NAT). Besides medical applications, NAT has been used in industry for preserving foods and crops. In this study, we investigated two parameters (pH and food ingredients) affecting NAT efficacy. In the human pathogen, *Aspergillus fumigatus*, NAT (2 to 16 μg mL^−1^) exerted higher activity at pH 5.6 than at pH 3.5 on a defined medium. In contrast, NAT exhibited higher activity at pH 3.5 than at pH 5.6 against foodborne fungal contaminants, *Aspergillus flavus*, *Aspergillus parasiticus*, and *Penicillium expansum*, with *P. expansum* being the most sensitive. In commercial food matrices (10 organic fruit juices), food ingredients differentially affected NAT antifungal efficacy. Noteworthily, NAT overcame tolerance of the *A. fumigatus* signaling mutants to the fungicide fludioxonil and exerted antifungal synergism with the secondary metabolite, kojic acid (KA). Altogether, NAT exhibited better antifungal activity at acidic pH against foodborne fungi; however, the ingredients from commercial food matrices presented greater impact on NAT efficacy compared to pH values. Comprehensive determination of parameters affecting NAT efficacy and improved food formulation will promote sustainable food/crop production, food safety, and public health.

## 1. Introduction

One Health recognizes that people, animals, plants, and their shared environment are highly interconnected. While recently One Health has focused the interface between human and animals, especially emerging infectious diseases, the scope of One Health also includes the prevention of foodborne diseases and contamination, noninfectious diseases, and environmental (agricultural) and ecosystem health [1]. For instance, invasive aspergillosis (IA) is a life-threatening infection caused by filamentous fungi in the genus *Aspergillus*. The most prevalent causative agent for IA is *Aspergillus fumigatus*, followed by *Aspergillus flavus*, *Aspergillus niger*, and *Aspergillus terreus* [2,3]. Immunocompromised people or patients are at a higher risk of developing fungal diseases [4]. Notably, besides human–animal interface, invasive aspergilli infection could also be acquired from contaminated foods (*A. fumigatus*) or crops (*A. flavus*), indicating human infections, such as IA, also involves food safety and environmental (agricultural) health issues [5]. However, control of fungal diseases is increasingly problematic due to the limited number of effective drugs/fungicides available for antifungal intervention [6]. Especially, cross-resistance of fungi to azole and polyene drugs has become a prominent public and environmental health issue [7]. Azoles, such as voriconazole (VRZ), itraconazole (ITR), or posaconazole (POS), are broadly used for treating human fungal pathogens. The primary mechanism of antifungal action of azoles is to inhibit the lanosterol 14-α sterol demethylase (CYP51 of the cytochrome P450 monooxygenase (CYP) superfamily) involved in fungal ergosterol biosynthesis [8,9]. Polyene drugs, such as amphotericin B (AMB), nystatin (NYS), and natamycin (NAT) (also known as pimaricin), contain conjugated double bonds in the structure, providing high affinity to ergosterol moiety of membranes in most fungal species [10].

Sequential combination antifungal therapy is sometimes necessary for effective control of fungi [7]. However, as observed in the yeast pathogens [11], prior exposure of *A. fumigatus* to azoles has been shown to lower the susceptibility of the fungal biofilms or germlings to polyenes such as AMB [12]. Of note, *A. fumigatus* pre-exposure to VRZ activated the cellular stress defense (namely adaptive resistance mechanisms) via the heat-shock protein 90 (Hsp90), which subsequently conferred AMB resistance, whereas pharmacological inhibition of Hsp90 by geldanamycin significantly enhanced (4- to 8-fold) the AMB susceptibility of fungal biofilm [12]. Hsp90 is a chaperone of calcineurin, a conserved Ca^2+^-calmodulin (CaM) activated protein phosphatase 2B, involved in the calcium-dependent signaling pathway for fungal virulence and stress response [13]. Therefore, studies indicated that azole-potentiated fungal tolerance to AMB is exerted via Hsp90-mediated oxidative stress defense [14]. We also determined previously that the cellular antioxidant system was a viable target in the antifungal action of azoles or AMB, where the mitochondrial superoxide dismutase (Mn-SOD), mitogen-activated protein kinases (MAPKs), etc., play crucial roles in fungal defense against azole- or AMB-induced oxidative stress [15,16].

Azoles are also increasingly applied in agricultural industry to control phytopathogenic fungi [17]. However, an azole-resistant *A. flavus* has been identified, where the T788G mutation in the *cyp51C* gene conferred the azole resistance [18]. *A. flavus* contaminates various agricultural commodities (during pre- and post-harvest) by producing the carcinogenic mycotoxins aflatoxins (AFs) [19]. Mutants of the other AF-producer *Aspergillus parasiticus*, having the G54W mutation in the *cyp51A* gene, produced AFs at concentrations significantly higher (up to 3-fold) than the wild-type strain [20]. Currently, more than 25% of total fungicide sales are azoles such as propiconazole or tebuconazole [17]. Therefore, once incorporated into the food supply chain, the azole-treated fungi, such as AF-overproducing *cyp51* mutants, will not only debilitate public food safety, but also cause significant economic losses, especially when the fungi are exposed subsequently to polyene fungicides during food production.

**Figure 1:**
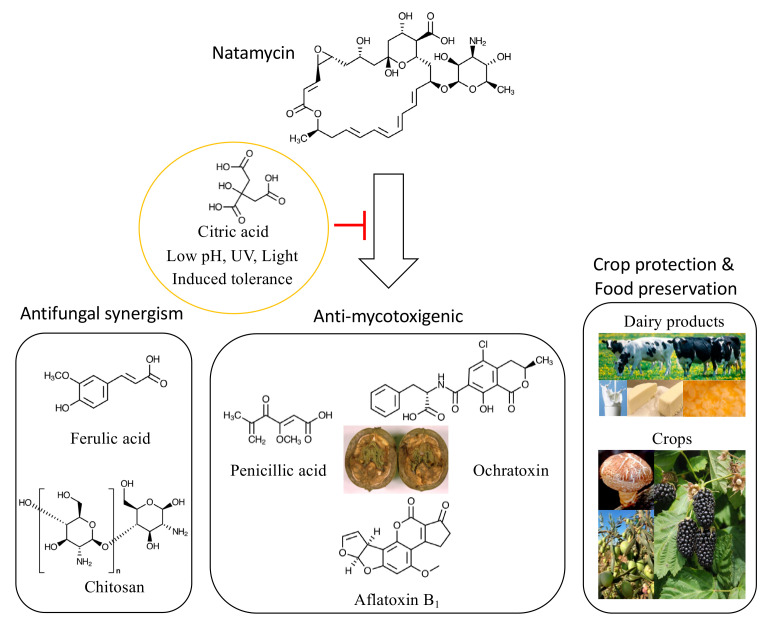
Natamycin usage in agricultural and food industries for crop protection, food preservation, and mycotoxin prevention (courtesy: USDA; see Table S1). Note that pH, light, UV, and food ingredients greatly affect the efficacy of NAT.

Among polyene molecules, NAT has been used widely for control of agricultural fungi infecting various crops (mushroom, tomato, strawberry, etc.) or contaminating processed foods (table olives, fruit juices, cheeses, yogurt, wines, etc.) (Figure 1; Table S1). For example, NAT was applied for postharvest disease control against *Botrytis cinerea* (to protect mandarin fruit), especially for managing isolates resistant to fungicides with different modes of action [21]. NAT also effectively controlled quinone outside inhibitor (QoI)-resistant *Colletotrichum acutatum* triggering strawberry crown rot, where the disease severity and plant mortality in field were reduced while fruit yield was significantly increased [22]. NAT also exhibited a synergistic antifungal activity with other compounds such as ferulic acid (FA), where coapplication of NAT and FA effectively inhibited the growth of *Aspergillus japonicus* and *Gilbertella persicaria* contaminating blackberry fruits [23]. Meanwhile, NAT has also exerted anti-mycotoxigenic capability; NAT effectively disrupted ochratoxin production by strains of *Aspergillus carbonarius* in grape juice-based medium [24]. In the baseline estimation of the postharvest pathogens, *Penicillium* sp., mean effective NAT concentrations for inhibiting 50% mycelial growth were determined as 1.54 and 1.14 μg mL^−1^ for *Penicillium digitatum* and *Penicillium expansum*, respectively [25]. Noteworthily, organic acids, such as lactic or citric acids, functioned as sensitizing agents to NAT in the green mold, *P. digitatum*, which resulted in a significant reduction in green mold contamination in lemon fruit [26]. In the food spoilage fungus, *Penicillium discolor*, NAT exhibited a different mode of action than other polyenes such as NYS; while NYS disrupted fungal plasma membrane, NAT could not permeabilize germinating conidia but inhibited their endocytosis [27].

Since fungicide resistance is an emerging environmental/public health concern [28], there is also an increasing demand for the development of precision NAT application practices, ensuring effective fungal control in the environment or food/crop production process. Studies have shown that NAT efficacy was affected greatly by pH, light, UV, and food ingredients such as salts or organic acids [29,30]. In this study, we investigated (1) redox-active characteristic of NAT; (2) differential antifungal efficacy of NAT between human pathogen (*A. fumigatus*) and mycotoxigenic, foodborne fungi (*A. flavus, A. parasiticus, Penicillium expansum*, *Neosartorya fischeri*) under two different pH conditions (3.5, high acidity; 5.6, low acidity), which mimic the conditions of commercial food matrices; and (3) impact of commercial food ingredients on NAT efficacy by using 10 organic fruit juices as experimental food matrices. 

## 2. Materials and Methods

### 2.1. Microorganisms

Fungal strains used in this study are described in Table 1. The human pathogen *A. fumigatus* (wild type and mitogen-activated protein kinase (MAPK) gene deletion mutants) and food-contaminating fungi *A. flavus* (aflatoxin (AF) producer)*, A. parasiticus* (AF producer), and *N. fischeri* (heat-resistant food spoilage strain) (Table 1) were maintained at 35 °C on potato dextrose agar (PDA; BD Life Sciences, Franklin Lakes, NJ, USA). *P. expansum* (wild type and fludioxonil (FLU)-resistant mutant; patulin producer) were grown at 28 °C on PDA.

### 2.2. Chemicals

All chemicals and media, namely polyenes (amphotericin B (AMB), nystatin (NYS), natamycin (NAT)), fludioxonil (FLU), kojic acid (KA), potato dextrose broth (PDB), and potato dextrose agar (PDA), were purchased from Sigma Aldrich Co., St. Louis, MO, USA. Dimethyl sulfoxide (DMSO) was procured from AMRESCO Co., Solon, OH, USA.

### 2.3. Overcoming FLU Tolerance of A. fumigatus MAPK Mutants (sakAΔ, mpkCΔ) by NAT

The capability of NAT to overcome FLU tolerance of *A. fumigatus* MAPK mutants was tested in PDA. NAT (2, 4, 6, or 8 μg mL^−1^) was incorporated into PDA without or with FLU (50 μM). Plates prepared were (a) wild type (AF293) and MAPK mutants with no treatment (Control), (b) NAT (2, 4, 6, or 8 μg mL^−1^), (c) FLU (50 μM), and (d) FLU (50 μM) + NAT (2, 4, 6, or 8 μg mL^−1^). Then, *A. fumigatus* spores (1 × 10^3^ CFU in 20 μL) were spotted at the center of the surface of each PDA (triplicate for each strain per test condition), and the inoculated plates were incubated at 35 °C. The level of fungal growth (% radial growth), including escaping of *sakA*Δ and *mpkC*Δ MAPK mutants from FLU toxicity, on each plate was monitored for 5 to 7 days, as described previously [34]. Compounds were dissolved in DMSO (absolute DMSO amount: <2% in media) before incorporation into culture media, and control plates contained DMSO only at levels equivalent to that of cohorts receiving NAT, within the same set of experiments.

### 2.4. Effect of pH (3.5 and 5.6) on the Antifungal Efficacy of NAT, AMB, or NYS

Differential antifungal efficacy of NAT, AMB, or NYS affected by different pHs (3.5, 5.6) was determined on PDA. Initially, PDB was dissolved in double-distilled water, then pHs were adjusted to 3.5 or 5.6. Select agar (1.5%) (Sigma Aldrich Co., St. Louis, MO, USA) was added to the pH-adjusted PDB, and then the resulting PDA was autoclaved at 121 °C for 25 min. The sterilized PDA (pH 3.5 or 5.6) was cooled down at room temperature, and NAT, AMB, or NYS (2, 4, 8, 16 μg mL^−1^) was incorporated into the PDA prepared. Then, the PDA was dispensed into Petri plates (60 mm × 15 mm; Corning Inc. Life Sciences, Tewksbury, MA, USA) (triplicate for each strain per test condition). Fungal spores (1 × 10^3^ CFU in 20 μL as determined by hemocytometer counts) from seven *Aspergillus*, two *Penicillium*, or one *Neosartorya* species were spotted onto the center of PDA. Fungal radial growth was determined as described above [34]. NAT, AMB, or NYS was dissolved in DMSO (absolute DMSO amount: <2% in media) before incorporation into culture media, and control plates (no NAT, no AMB, no NYS) contained DMSO only at levels equivalent to that of cohorts receiving antifungal agents, within the same set of experiments. Fungal growth (% radial growth) was monitored for 5 to 7 days at 35 °C, except *P. expansum* which was grown at 28 °C.

### 2.5. Antifungal Synergism (Chemosensitization) between NAT and KA

Chemosensitizing activity (antifungal synergism) of KA to NAT was determined against *A. fumigatus* wild type and MAPK mutants using PDA bioassay. PDA plates (pH 5.6) supplemented with NAT (1, 2, 3 μg mL^−1^), without or with 5 mM KA, were prepared in triplicate per strain, and fungal spores (1 × 10^3^ CFU in 20 μL) were spotted at the center of each PDA, as described above. The inoculated plates were incubated at 35 °C, and fungal growth (% radial growth) was monitored for 5 to 7 days. Compounds were dissolved in DMSO (absolute DMSO amount: <2% in media) before incorporation into culture media, and control plates (no NAT and/or no KA) contained DMSO only at levels equivalent to that of cohorts receiving antifungal agents, within the same set of experiments.

### 2.6. Effect of Food Ingredients on the Efficacy of NAT Tested on Organic Fruit Juice Agar 

Organically produced fruit juices were purchased from local grocery stores (a total of 10 fruit juices as follows: #1—white grape (pH 3.08), #2—honey crisp apple (pH 3.48), #3—blueberry (pH 3.17), #4—black cherry (pH 3.99), #5—tart cherry (pH 3.41), #6—concord grape (pH 3.59), #7—grapefruit (pH 3.21), #8—pear (pH 3.95), #9—pineapple (pH 3.95), #10—pomegranate (pH 3.36)) (Berkeley, CA, USA). No artificial preservatives were commercially added into the fruit juices by the producers (compositions not disclosed; trade secret). Initially, pHs of each fruit juice were measured, and then each juice was supplemented with 1.5% select agar (Sigma Aldrich Co., St. Louis, MO, USA) in 500 mL glass media bottles (Corning Inc. Life Sciences, Tewksbury, MA, USA). All preparations were autoclaved at 121 °C for 25 min. The sterilized juice agar (JA) (pHs 3.08 to 3.99; equivalent to each fruit juice) was cooled down at room temperature, and NAT (2, 4, 6, 8 μg mL^−1^) was incorporated into the JA. Then, JA was dispensed into Petri plates (60 mm × 15 mm; Corning Inc. Life Sciences, Tewksbury, MA, USA) (triplicate for each strain per test condition). Fungal spores (1 × 10^3^ CFU in 20 μL as determined by hemocytometer counts) from seven *Aspergillus*, two *Penicillium*, or one *Neosartorya* species were spotted onto the center of JA. Fungal growth (% radial growth) was monitored for 5 to 7 days at 35 °C, except *P. expansum* which was grown at 28 °C, as described above. NAT was dissolved in DMSO (absolute DMSO amount: <2% in media) before incorporation into culture media, and control plates (no NAT) contained DMSO only at levels equivalent to that of cohorts receiving NAT, within the same set of experiments. We initially described the NAT antifungal efficacy in radial growth (%), and then the values were translated further into the rank of each fruit juice (see Section 3.2.2). Antifungal ranks were expressed as “1” (highest antifungal activity) to “10” (lowest antifungal activity) at each concentration (2, 4, 8, or 16 μg mL^−1^) of NAT in 10 organic fruit juices.

### 2.7. Statistical Analysis

Statistical analysis (Student’s *t*-test) was performed based on “Statistics to use” [35], where *p* < 0.05 was considered significant.

## 3. Results and Discussion

### 3.1. Redox Activity of NAT: Overcoming FLU Tolerance of A. fumigatus Antioxidant Signaling Mutants

**Figure 2:**
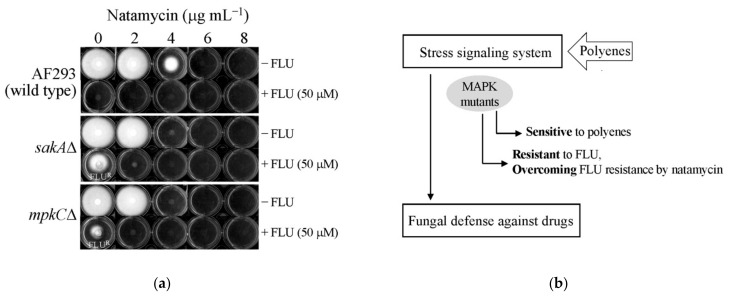
(**a**) The level of fungal growth without or with the treatment of natamycin (NAT) (2, 4, 6, 8 μg mL^−1^) and/or fludioxonil (FLU) (50 μM) tested in *A. fumigatus* AF293 (wild type) and MAPK mutants (*sakA*Δ, *mpkC*Δ); (**b**) diagram: overcoming FLU tolerance of *A. fumigatus* MAPK mutants by combined application of NAT.

The polyene drugs AMB and NYS have been shown to induce reactive oxygen species (ROS), thus triggering oxidative stress in fungal cells (planktonic free-living or on biofilm) [14]. However, the redox-active characteristic of NAT (as an antifungal agent) has not been documented so far. Noteworthily, in *Streptomyces natalensis* (Gram-positive, filamentous soil bacterium), an imbalance of the intracellular reactive oxygen species (ROS) homeostasis leads to the crosstalk between ROS homeostasis and secondary metabolism, thus modulating NAT production for countering the stress [36,37]. Results showed that *A. fumigatus* antioxidant signaling mutants (*sakA*Δ, *mpkC*Δ) [31,32] were more sensitive to NAT than the wild type (AF293) (Figure 2). The growth of MAPK mutants was completely inhibited with NAT at ≥4 μg mL^−1^, while the wild type still maintained about 46% radial growth at 4 μg mL^−1^ of NAT. Results indicated that, like AMB or NYS, NAT is a also redox-active molecule, and therefore, fungal cells lacking the intact oxidative stress defense system exhibited increased susceptibility to NAT. SakA and MpkC are orthologous proteins to the high osmolality glycerol (HOG) MAPK protein (Hog1p) of the model yeast *Saccharomyces cerevisiae*, which is involved in oxidative stress signaling/defense [38]. *A. fumigatus sakA*Δ and *mpkC*Δ mutants previously showed enhanced susceptibility to the redox-active benzo derivatives [34].

Fungi having mutations in the antioxidant system escape toxicity of the commercial fungicide FLU [39]. *A. fumigatus sakA*Δ and *mpkC*Δ also exhibited tolerance to 50 μM FLU, resulting in maintenance of 40 to 60% radial growth, respectively, compared to the wild type control (no growth). However, coapplication of sublethal concentration of NAT (2 μg mL^−1^) with FLU (50 μM) resulted in an effective fungal control; the coapplication did not allow MAPK mutants to develop tolerance to FLU, thus achieving complete fungal death (Figure 2). 

FLU is a phenylpyrrole fungicide that triggers excessive activation of the intact MAPK system for stress defense, such as glycerol biosynthesis [39]. The excessive activation of MAPK thus results in an energy drain and induction of osmotic/oxidative imbalance in fungal cells that eventually disrupts the growth of fungal pathogens. We reasoned that the redox-active NAT directly targets the downstream of MAPK signaling system in fungi [40,41], for which the structural genes in the antioxidant system, such as superoxide dismutases (cytosolic or mitochondrial) and glutathione homeostasis, are responsible for fungal defense against NAT. However, with the MAPK mutation, these antioxidant systems cannot be expressed/operated in the cell, thus resulting in enhanced susceptibility of fungi to the NAT treatment.

### 3.2. Differential NAT Susceptibility between Human Pathogen (A. fumigatus) and Foodborne Fungal Contaminants (A. flavus, A. parasiticus, P. expansum, N. fischeri) under Different pHs: High (pH 3.5) versus Low (pH 5.6) Acidity Conditions

#### 3.2.1. Fungal Susceptibility to Polyene Drugs (NAT, AMB, NYS): PDA Test

**Figure 3:**
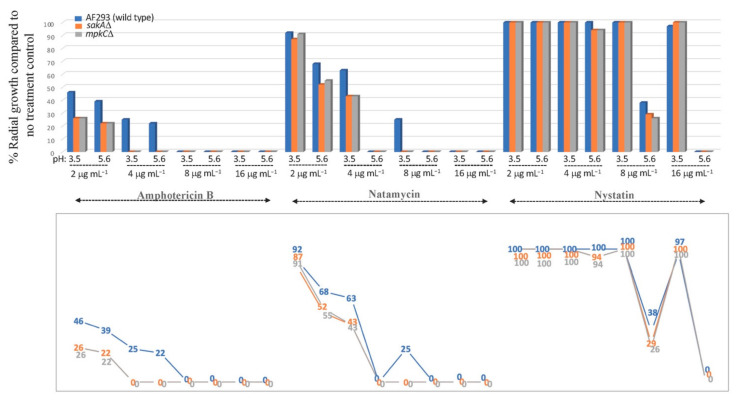
Susceptibility of *A. fumigatus* AF293 (wild type) and MAPK mutants (*sakA*Δ, *mpkC*Δ) to polyene drugs (amphotericin B (AMB), natamycin (NAT), nystatin (NYS); 2, 4, 8, 16 μg mL^−1^) tested in PDA (triplicate) at pH 3.5 or 5.6 (SD < 2%). Lower panel: Line graph showing numerical values of % radial growth at each test condition. (Note: While NAT showed higher antifungal activity than AMB at 4 μg mL^−1^ (pH 5.6), where NAT completely inhibited the growth of AF293, the 22% growth with AMB labeled in the graph indicated the appearance of scattered colonies only (not a full radial growth) of AF293.)

Antifungal efficacy of NAT is greatly influenced by pH [29,30]. The level of susceptibility of *A. fumigatus* to all three polyene drugs was higher at low acidity (pH 5.6) than at high acidity (pH 3.5) condition, where AMB showed the highest antifungal activity (order of antifungal activity, high to low: AMB > NAT > NYS) (Figure 3). Moreover, the MAPK mutants exhibited higher susceptibility to NAT compared to the wild type (AF293); thus, as determined in AMB and NYS, results confirmed further the redox-active characteristic of NAT (see also Figure 2).

Regarding NAT, all *A. fumigatus* strains tested exhibited higher NAT susceptibility (37 to 57% less radial growth) at pH 5.6 than at pH 3.5 (Figure 3). Notably, the growth of *A. fumigatus* strains was completely inhibited by as low as 4 μg mL^−1^ of NAT at pH 5.6, while the growth of AF293 still reached 25% even at 8 μg mL^−1^ of NAT at pH 3.5, highlighting the importance of lower acidic condition for the optimum NAT efficacy against *A. fumigatus*. Similar to NAT, the growth of *A. fumigatus* AF293 and MAPK mutants still reached 97 to 100% with 16 μg mL^−1^ (the highest concentration tested) of the other polyene NYS at pH 3.5, while that with 8 μg mL^−1^ NYS reached 26 to 38% at pH 5.6, thus achieving much higher fungal control (namely 59 to 71% less growth) even at the lower dose of NYS at pH 5.6 (Figure 3).

**Figure 4:**
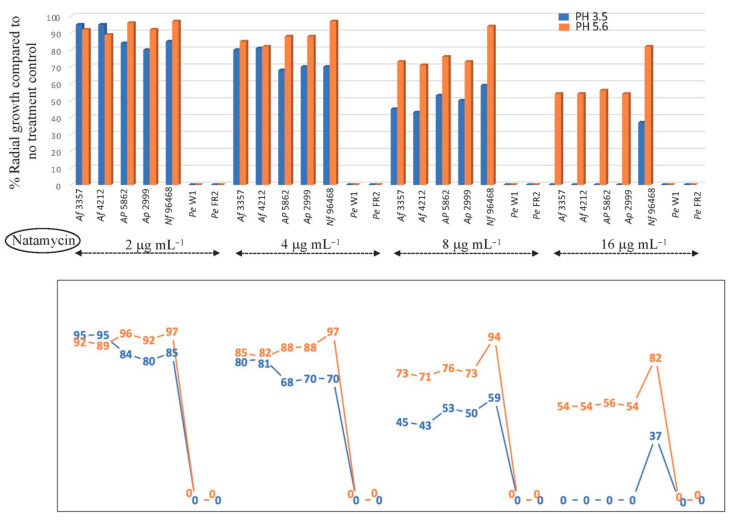
Susceptibility of foodborne fungal contaminants (*Af*, *A. flavus*; *Ap, A. parasiticus*; *Nf, N. fischeri*; *Pe*, *P. expansum*) to natamycin (NAT) (2, 4, 8, 16 μg mL^−1^) tested in PDA at pH 3.5 or 5.6 (triplicate) (SD < 2%). Lower panel: Line graph showing numerical values of % radial growth at each test condition.

Surprisingly, the antifungal efficacy of NAT against foodborne fungi was much higher (12 to 56% reduction in radial growth) at pH 3.5 than at pH 5.6 at all NAT concentrations investigated (Figure 4), which is contrary to the *A. fumigatus* results. The only exception was *A. flavus* NRRL 3357 and NRRL 4212 at 2 μg mL^−1^ of NAT, where NAT exerted slightly higher antifungal activity (3 to 6% less radial growth) at pH 5.6 when compared to that at pH 3.5. The level of NAT susceptibility of *A. flavus* NRRL 3357 and NRRL 4212 was also relatively low at 4 μg mL^−1^ of NAT, where NAT exerted slightly higher antifungal activity (1 to 5% less radial growth) at pH 3.5 than at pH 5.6. Other foodborne fungi (*A. parasiticus*, *N. fischeri*, *P. expansum*) exhibited much higher susceptibility to NAT (18 to 100% less radial growth) under the same test conditions (Figure 4). Therefore, results indicated that, although *A. flavus* NRRL 3357 and NRRL 4212 were slightly less susceptible to the lower doses of NAT (up to 4 μg mL^−1^), all fungi including both *A. flavus* NRRL 3357 and NRRL 4212 showed 23 to 100% less growth at pH 3.5 than at pH 5.6 with higher doses (8 to 16 μg mL^−1^) of NAT, thus achieving effective fungal control.

Altogether, the trends of fungal susceptibility to NAT determined in foodborne fungal contaminants were contrary to *A. fumigatus* results. *P. expansum* showed the highest susceptibility to NAT (i.e., fungal death at all concentrations of NAT tested), while *A. flavus* NRRL 3357 and NRRL 4212 were less susceptible to NAT at lower concentrations (up to 4 μg mL^−1^) compared to other foodborne fungi (Figure 4). Rusul and Marth [29] previously reported that, in a culture medium with pH 3.5, no growth or no mycotoxin production could be determined in *A. parasiticus* treated with >7.5 μg mL^−1^ of NAT (also known as pimaricin), which is similar to our observation. In general, the level of fungal susceptibility to NAT (at the respective optimum pH) was higher in *A. fumigatus* than in *A. flavus* or *N. fischeri*; for instance, at 4 μg mL^−1^ NAT, *A. fumigatus* showed complete growth inhibition (pH 5.6) while *A. flavus* or *N. fischeri* exhibited 19 to 32% growth inhibition (pH 3.5) (Figure 3 and Figure 4).

We speculate that one of the mechanisms of the increased NAT efficacy at low pH against foodborne fungi could be the protonation of a nitrogen in the NAT structure to a quaternary form (NH_4_^+^); however, the mechanism of reversed effects observed in the human pathogen *A. fumigatus* is not clear. The effects of pH on differential antifungal activity of drugs/preservatives have been documented previously (Table S2). For example, *A. fumigatus* exhibited higher susceptibility to the antimalarial drug chloroquine (CQ) than *Aspergillus nidulans* at pH 8 [42]. However, no significant differences in CQ antifungal efficacy were found between *A. fumigatus* and *A. nidulans* at pH 6. It has been determined that a higher extracellular pH (pH 8) enabled increased diffusion of CQ into the target fungi [42], whereas the antifungal activity of 5-flucytosine (5-FC), a structural derivative of the nucleobase cytosine, against *A. fumigatus* was determined to be lower at pH 7 than at pH 5 [43]. Molecular analysis identified that the *A. fumigatus fcyB* gene encoding a purine-cytosine permease, an orthologous 5-FC importer, was downregulated at pH 7, thus negatively affecting 5-FC uptake into fungi [43]. In the AF-producing *A. flavus* and *A. parasiticus*, the food preservatives potassium sorbate, sodium sulfite, and sodium propionate exhibited high antifungal activity at pH 3, while that determined at pHs 4.5 to 7.0 was low [44,45].

The differential antifungal efficacy of drugs/compounds at various pHs was also investigated in the yeast-form pathogens. Valproic acid (VPA) is an antipsychotic drug recently repurposed to treat *Candida albicans* [46]. VPA exhibited high anticandidal activity at acidic pH (pH 4.5), while that determined at pH 8.0 was low. The mechanism of increased antifungal action at acidic pH was the alteration of vacuole integrity by VPA [46]. More examples of drugs/compounds exhibiting differential antifungal activity at acidic or basic pHs have been reported (See Table S2). Differential cellular uptake, diffusion of molecules, modified organellar integrity, etc., have been the proposed mechanisms of pH-dependent high or low antifungal efficacy. Determination of the precise mechanism of differential NAT susceptibility between *A. fumigatus* and foodborne fungal contaminants at different pHs, namely high versus low acidity conditions, warrants future in-depth investigation.

#### 3.2.2. Fungal Susceptibility to Polyene Drugs (NAT, AMB, NYS): Food Matrices (Organic Fruit Juices) Test

We observed in the *A. fumigatus* test that, although NAT antifungal efficacy was generally enhanced with increasing concentrations of NAT in all fruit juices tested, the level of antifungal efficacy of NAT varied depending on the types of fruit juices or fungi examined (Table 2; Figures S1 and S2). 

In Table 3, while the “average” rank of each fruit juice was calculated from the values from all NAT concentrations (2, 4, 8, or 16 μg mL^−1^), the “final” rank of each fruit juice was determined by ordering them by average rank (Table 3). The final rank reflects the “expected” relative NAT efficacy in each fruit juice examined. 

Generally, the relative rank (antifungal) of each fruit juice depended on the types of juices and NAT concentrations applied. Key observations with the human pathogen *A. fumigatus* are as follows: (1) The ranks of fruit juices 9 and 10 lowered (e.g., rank “1” to “10”; juice 10) as the NAT concentration increased (2 to 16 μg mL^−1^) (Table 3), whereas the relative ranks of juices 5 and 8 improved (e.g., rank “5” to “2”; juice 8) as the NAT concentration increased (Table 3). (2) Juice 7 exhibited very low antifungal efficacy at 2, 4, and 8 μg mL^−1^ of NAT (namely rank “10”) compared to that determined in other fruit juices. Surprisingly, complete growth inhibition of all *A. fumigatus* (AF293, MAPK mutants) was achieved at 16 μg mL^−1^ NAT (rank “1”) (Table 2 and Table 3; Figure S1). Such contrasting NAT efficacy between the low and high NAT concentrations indicated that juice 7 might contain ingredients possessing high redox-active characteristics. Redox-active compounds have both antioxidant and prooxidant potential [47] and therefore can serve as potent redox-cyclers in pathogens above the oxidative stress threshold level. While the redox-active molecules function as antioxidants at lower concentrations, thus countering the NAT-triggered oxidative stress in fungi, they could function as potent antimicrobials by disrupting cellular redox homeostasis and/or redox-sensitive structures in fungi [48,49]. We previously determined that lower levels (<3 mM) of the phenolic 2,5-dihydroxybenzoic acid (2,5-DHBA) countered the H_2_O_2_-triggered oxidative stress in the model yeast *Saccharomyces cerevisiae*, while the growth of the yeast was disrupted when treated with 3 to 18 mM (above the oxidative stress threshold level) of 2,5-DHBA [50]. We surmised that redox-active compounds, such as NAT or 2,5-DHBA, exerted heightened antifungal activity in the presence of additional redox-potent molecules (for example, the redox-active ingredients in fruit juice 7) in the food matrices. (3) Except for the 16 μg mL^−1^ NAT in juice 7 (0% growth), complete fungal death could not be achieved with NAT in any of the fruit juices examined (Figure S1). Considering the similar pH values of fruit juices (average: pH 3.5; 3.08 to 3.99) to PDA (pH 3.5) where complete fungal death was achieved at 4 μg mL^−1^ NAT, we speculated that the ingredients in fruit juices have higher impact on NAT efficacy compared to pH. (4) AF293 generally exhibited higher NAT tolerance than MAPK mutants in fruit juices; an exception is that, in juice 9, the level of NAT efficacy against all *A. fumigatus* strains (AF293, MAPK mutants) remained similar at 4, 8, or 16 μg mL^−1^, regardless of NAT doses applied (namely 19% radial growth) (Figure S1). (5) In general, the trends of NAT antifungal efficacy against two MAPK mutants were similar within the same fruit juice matrices (Figure S1). For example, in juice 4, similar levels of MAPK (*sakA*Δ, *mpkC*Δ) susceptibility to NAT were determined, namely 33%, 33%, or 27% growth at 4, 8, or 16 μg mL^−1^ NAT, respectively (Figure S1). As observed in *A. fumigatus*, the relative rank (antifungal) of each fruit juice determined in foodborne fungal contaminants also depended on the types of juices and NAT concentrations applied. Key observations with the foodborne fungal contaminants are as follows: (1) The relative ranks of fruit juices 9 and 10 lowered (e.g., rank “2” to “6”; juice 10) as the NAT concentration increased (2 to 16 μg mL^−1^) (Table 3), whereas, the relative ranks of fruit juices 3 and 5 were improved (e.g., rank “7” to “2”; juice 5) as the NAT concentration increased (Table 3). (2) The NAT efficacy was determined high in fruit juice 6 for foodborne fungal contaminants (similar to *A. fumigatus*, namely rank “1” or “2” for foodborne fungi or *A. fumigatus*, respectively) (Table 3). (3) In *A. fumigatus*, none of NAT treatments in any of the fruit juices could completely inhibit fungal growth (compared to PDA test; see Figure 4), except for 16 μg mL^−1^ NAT in “juice 7” which achieved 0% radial growth in *P. expansum* W1 and FR2 (Figure S2). Results confirmed further that food ingredients have more impact on NAT antifungal efficacy than pH (see *A. fumigatus* results above). Notably, *P. expansum* and *A. fumigatus* exhibited similar susceptibility to NAT in juice 7, indicating these fungi might possess similar response mechanism to the NAT in fruit juice 7. (4) The NAT susceptibilities of the AF-producing fungi (*A. flavus*, *A. parasiticus*) were determined similar in all fruit juices examined (Figures S3 and S4). Of note, the NAT antifungal efficacy in juice 4 was determined “low” in most of *A. fumigatus* and foodborne fungal contaminants (Figures S1–S4).

The antifungal efficacy of NAT in response to food ingredients has been investigated previously in different food matrices. For instance, in simulated acid sauces, NAT at 12 mg L^−1^ effectively inhibited the growth of the food-contaminating yeast *Zygosaccharomyces bailii* [51]. However, combined application of 3.00 to 6.00% of NaCl resulted in antagonism between NAT and NaCl, thus lowering the antifungal efficacy of NAT. Moreover, addition of one of the commercial food ingredients, xanthan gum (XG), at 0.25 to 0.50% also decreased NAT efficacy. Therefore, the study proved the importance of selecting proper ingredients during food formulation when NAT is coapplied [51].

In table olive matrices, citric acid at lower than 0.15% decreased the antifungal efficacy of NAT against three yeast species, *Saccharomyces cerevisiae*, *Wickerhamomyces anomalus*, and *Candida boidinii*, which commonly contaminate table olives [52]. Similar to acid sauce results (above), NaCl at around 5% also enhanced NAT resistance of *S. cerevisiae* and *C. boidinii*, thus indicating further the antagonistic effect of certain food ingredients against NAT in commercial food matrices [52].

Antifungal efficacy of NAT has been compared between PDA and cheese matrices (pH 5.0) against many fungi, including Mucor racemosus, Penicillium commune, Galactomyces geotrichum, Yarrowia lipolytica, Phoma pinodella, Candida parapsilosis, Meyerozyma guilliermondii, Trichosporon asahii, and Rhodotorula mucilaginosa [53]. Remarkably, compared to other food matrices studies described above, NAT activity was determined higher on cheese surfaces compared to that on PDA. Possible mechanisms of enhanced NAT activity were speculated to be (1) the synergism exerted by the molecules in the cheese, such as lactic acid, produced by starter cultures and (2) the differences in the physiological status of the target fungi in different matrices [53].

### 3.3. Enhanced Antifungal Activity of Polyenes with KA in Filamentous Fungi

Antifungal chemosensitization is a new intervention strategy, where combined application of a second molecule (chemosensitizer; either natural or synthetic) with a conventional drug/fungicide could enhance the antifungal efficacy of the coapplied drug/fungicide [54]. The key value of chemosensitization is that, compared to traditional combination therapy which is a coapplication of two or more conventional antifungal drugs/fungicides, a chemosensitizer itself does not have to possess a potent antifungal activity. A chemosensitizer causes the target fungi to become more susceptible to the commercial drug/fungicide coapplied by modulating fungal defense system, such as antioxidant pathway, to the drug/fungicide [54]. 

**Figure 5:**
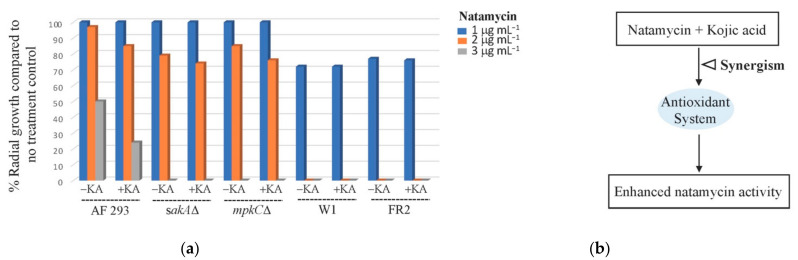
(**a**) The level of fungal growth without or with the treatment of natamycin (NAT) (1, 2, 3 μg mL^−1^) and/or kojic acid (KA) (5 mM) tested in *A. fumigatus* (AF293 (wild type), MAPK mutants (*sakA*Δ, *mpkC*Δ)) and *P. expansum* (W1 (wild type), FR2 (FLU-resistant mutant)) (triplicate) (SD < 2%); (**b**) diagram: enhanced NAT activity by combined application of KA.

Kojic acid (KA; 5-hydroxy-2-(hydroxymethyl)-4H-pyran-4-one) is a natural pyrone produced mainly by the species of *Aspergillus* and *Penicillium* [55]. We previously determined that KA exerted chemosensitizing antifungal activity to AMB (synergism) against *A. fumigatus* by lowering minimum inhibitory (MIC) or fungicidal (MFC) concentrations of AMB [55]. In the present study, we determined the chemosensitizing activity of KA to NAT against *A. fumigatus* AF293, *sakA*Δ, and *mpkC*Δ by coapplying KA (5 mM) with 1, 2, or 3 μg mL^−1^ of NAT. We observed that the level of growth inhibition of *A. fumigatus* was increased 5 to 26%, depending on the types of strains (Figure 5). However, the level of growth inhibition by KA + NAT was not comparable to that exerted by KA + AMB coapplication, where the MAPK mutants exhibited 90.0 to 99.9% fungal death with 0.4 mM KA + 2 μg mL^−1^ AMB (previously determined by Kim et al. [55]), thus reflecting the higher antifungal potency of AMB compared to NAT.

## 4. Summary and Conclusions

In this study, we investigated parameters that affect the antifungal efficacy of NAT in PDA (defined medium) and commercial food matrices (10 fruit juices). We identified the redox-active characteristic of NAT, which overcame the FLU tolerance of fungal mutants lacking key genes in the oxidative stress signaling system. In PDA, NAT exerted higher antifungal activity at pH 5.6 than at pH 3.5 against *A. fumigatus*, while that determined in foodborne fungi exhibited reversed trends. Protonation of nitrogen in NAT (NH_4_^+^), differential cellular uptake, diffusion efficacy of molecules, up- or downregulation of genes encoding membrane transporters, etc., could affect NAT efficacy at different pHs. In the commercial food matrices, the ingredients from individual juices had a greater impact on NAT efficacy compared to pH values. Despite the moderate enhancement, antifungal synergism between NAT and KA during chemosensitization has been confirmed, which could serve as an effective tool or strategy for improved food formulation. Altogether, our results provide information for the precision NAT application practices. Comprehensive identification of parameters that affect NAT efficacy in food preservation, elucidation of the mechanism of action, and improved food formulation will promote sustainable food and crop production, thus warranting food safety and public health.

## Figures and Tables

**Table 1:** Characteristics of fungi used in this study.

Fungi	Strain Characteristics	Source
*A. flavus* 3357	Mycotoxigenic (aflatoxin (AF) producer), human pathogen (aspergillosis), reference strain for genome sequencing	NRRL ^1^
*A. flavus* 4212	Mycotoxigenic (AF producer), human pathogen (aspergillosis)	NRRL
*A. parasiticus* 2999	Mycotoxigenic (AF producer)	NRRL
*A. parasiticus* 5862	Mycotoxigenic (AF producer)	NRRL
*A. fumigatus* AF293	Human pathogen (aspergillosis), reference clinical strain for genome sequencing	UT ^2^, [31]
*A. fumigatus sakA*Δ	Mitogen-activated protein kinase (MAPK) gene deletion mutant derived from AF293	UT, [31]
*A. fumigatus mpkC*Δ	MAPK gene deletion mutant derived from AF293	UT, [32]
*P. expansum* W1	Mycotoxigenic (patulin producer; parental strain)	WSU ^3^, [33]
*P. expansum* FR2	Fludioxonil (FLU)-resistant mutant derived from W1	WSU, [33]
*N. fischeri* 96468	Food spoilage fungus, heat resistant	ATCC ^4^

^1^ NRRL, National Center for Agricultural Utilization and Research, USDA-ARS, Peoria, IL, USA. ^2^ UT, The University of Texas, MD Andersen Cancer Center, Houston, TX, USA. ^3^ WSU, Washington State University, Wenatchee, WA, USA. ^4^ ATCC, American Type Culture Collection, Manassas, VA, USA.

**Table 2:** Levels of fungal susceptibility to NAT in different fruit juices. ^1^

NAT (μg mL^−1^)	Values	PDA (pH 3.5)	Juice 1	Juice 2	Juice 3	Juice 4	Juice 5	Juice 6	Juice 7	Juice 8	Juice 9	Juice 10
*A. fumigatus*	Average	90.0	86.3	72.3	77.0	77.7	79.3	67.3	90.0	72.7	66.7	54.7
2	SD	2.6	5.8	2.3	7.0	4.0	4.0	3.1	0.0	2.5	6.7	10.8
	*p*-value	-	0.37	0.00 *	0.04 *	0.01 *	0.02 *	0.00 *	1.00	0.00 *	0.01 *	0.01 *
4	Average	49.7	62.3	41.7	45.3	39.7	52.0	24.3	81.7	19.3	19.0	33.0
	SD	11.5	5.8	2.3	8.5	11.5	3.5	7.5	2.3	5.1	0.0	14.7
	*p*-value	-	0.16	0.31	0.63	0.35	0.75	0.03 *	0.01 *	0.01 *	0.01 *	0.20
8	Average	8.3	33.3	19.0	24.3	33.0	16.7	15.7	49.3	10.3	19.0	33.0
	SD	14.4	6.8	1.7	2.1	0.0	2.9	0.6	5.1	2.3	0.0	14.7
	*p*-value	-	0.05	0.27	0.13	0.04 *	0.38	0.43	0.01 *	0.82	0.27	0.11
16	Average	0.0	18.0	15.7	17.7	29.0	13.3	11.7	0.0	9.0	19.0	33.0
	SD	0.0	0.0	2.9	2.1	3.5	2.3	0.6	0.0	0.0	0.0	14.7
	*p*-value	-	0.00 *	0.00 *	0.00 *	0.00 *	0.00 *	0.00 *	ND	0.00 *	0.00 *	0.02 *
Foodborne fungi	Average	62.7	82.1	73.9	77.1	82.7	80.6	72.6	82.0	74.7	75.7	73.7
2	SD	43.2	19.4	19.0	13.4	22.4	13.0	17.0	14.0	22.8	18.1	8.4
	*p*-value	-	0.30	0.54	0.42	0.30	0.32	0.58	0.28	0.53	0.48	0.52
4	Average	52.7	73.6	56.4	56.4	77.6	57.4	52.4	59.0	60.7	65.6	55.4
	SD	36.4	22.1	14.5	14.3	19.1	20.2	13.1	40.5	19.2	14.4	12.9
	*p*-value	-	0.22	0.81	0.81	0.14	0.77	0.99	0.77	0.62	0.40	0.86
8	Average	35.7	66.4	42.3	40.6	67.3	42.7	36.7	50.4	46.9	52.6	41.4
	SD	25.0	18.3	7.0	10.0	13.3	9.4	7.4	35.2	10.6	8.6	14.3
	*p*-value	-	0.02 *	0.52	0.64	0.01 *	0.50	0.92	0.38	0.30	0.12	0.61
16	Average	5.3	55.7	30.3	30.1	54.1	26.6	22.1	40.6	33.4	39.3	35.1
	SD	14.0	12.7	8.4	10.6	6.6	7.9	10.4	28.1	7.8	9.5	17.0
	*p*-value	-	0.00 *	0.00 *	0.00 *	0.00 *	0.00 *	0.03 *	0.01 *	0.00 *	0.00 *	0.00 *

^1^ Average, average % radial growth calculated from three strains (AF293, *sakA*Δ, *mpkC*Δ) of *A. fumigatus* or seven strains (*A. flavus* 3357, *A. flavus* 4212, *A. parasiticus* 5862, *A. parasiticus* 2999, *N. fischeri* 96468, *P. expansum* W1, *P. expansum* FR2) of foodborne fungal contaminants; SD, standard deviation calculated from three strains of *A. fumigatus* or seven strains of foodborne fungal contaminants; ND, not determined; Student’s *t*-test for paired data (juices 1 to 10) was vs. the data of *A. fumigatus* or foodborne fungi determined in PDA (pH 3.5; *p*-value designation: “-“). *p*-values < 0.05 considered significant are marked with asterisks (*).

**Table 3:** Antifungal ranks of organic fruit juices reflecting the impact of each juice’s ingredients on the levels of NAT efficacy. ^1^

Juice No. NAT (μg mL^−1^)	1	2	3	4	5	6	7	8	9	10
Human pathogen (*A. fumigatus*)										
2	9	4	6	7	8	3	10	5	2	1
4	9	6	7	5	8	3	10	2	1	4
8	9	4	6	7	3	2	10	1	4	7
16	7	5	6	9	4	3	1	2	8	10
Average ^2^	8.50	4.75	6.25	7.00	5.75	2.75	7.75	2.50	3.75	5.50
Final ^3^	10	4	7	8	6	2	9	1	3	5
Foodborne contaminants										
2	9	3	6	10	7	1	8	4	5	2
4	9	3	3	10	5	1	6	7	8	2
8	9	4	2	10	5	1	7	6	8	3
16	10	4	3	9	2	1	8	5	7	6
Average ^2^	9.25	3.50	3.50	9.75	4.75	1.00	7.25	5.50	7.00	3.25
Final ^3^	9	3	3	10	5	1	8	6	7	2

^1^ Antifungal ranks are expressed as “1” (highest antifungal activity) to “10” (lowest antifungal activity) at each concentration (2, 4, 8, or 16 μg mL^−1^) of NAT in 10 fruit juices. ^2^ Average, average rank of each fruit juice calculated from the ranks determined in all concentrations (2, 4, 8, or 16 μg mL^−1^) of NAT. ^3^ Final, final rank of each fruit juice derived from the average rank exhibited above, which reflects the expected relative NAT efficacy added into each fruit juice.

## Data Availability

The data generated by this study are available in this paper.

## Supplementary Materials

The following are available online at https://www.mdpi.com/article/10.3390/foods10092073/s1, Figure S1: Susceptibility of *A. fumigatus* AF293 (wild type) and MAPK mutants (*sakA*Δ, *mpkC*Δ) to natamycin (NAT) (2, 4, 8, 16 μg mL^−1^) tested in the commercial, organic fruit juices (triplicate) (SD < 2%). Lower panel: Line graph showing numerical values of % radial growth at each test condition, Figure S2: Susceptibility of foodborne fungal contaminants (*Pe, P. expansum*; *Nf, N. fischeri*) to natamycin (NAT) (2, 4, 8, 16 μg mL^−1^) tested in the commercial, organic fruit juices (triplicate) (SD < 2%). Lower panel: Line graph showing numerical values of % radial growth at each test condition, Figure S3: Susceptibility of foodborne fungal contaminants (*A. flavus* NRRL 3357, NRRL 4212) to natamycin (NAT) (2, 4, 8, 16 μg mL^−1^) tested in the commercial, organic fruit juices (triplicate) (SD < 2%). Lower panel: Line graph showing numerical values of % radial growth at each test condition, Figure S4: Susceptibility of foodborne fungal contaminants (*A. parasiticus* NRRL 2999, NRRL 5862) to natamycin (NAT) (2, 4, 8, 16 μg mL^−1^) tested in the commercial, organic fruit juices (triplicate) (SD < 2%). Lower panel: Line graph showing numerical values of % radial growth at each test condition, Table S1: Utility of natamycin (NAT) in food and agricultural industries, Table S2: Examples of differential antifungal efficacy of drugs/preservatives affected by different pHs.
