# Supplementary material for: Antifungal Efficacy of Redox-Active Natamycin against Some Foodborne Fungi—Comparison with Aspergillus fumigatus
Source: Foods. 2021 Sep 2;10(9):2073. doi: 10.3390/foods10092073 (PMC8469148; doi:10.3390/foods10092073)
Supplement: Supplementary file 1 [file foods-10-02073-s001.zip › foods-1307195-supplementary.pdf]

SUPPLEMENTARY MATERIALS

Table S1. Utility of natamycin (NAT) in food and agricultural industries.

Test system	Fungi targeted	Effects	References
Anti-mycotoxigenic:			
Whole olives, olive paste samples	*Aspergillus flavus*	The growth level was parallel to the temperatures of incubation. The aflatoxin B_1 production in all samples at all levels of preservatives were negative.	[1]
Grape juice-based medium	*Aspergillus carbonarius*	Natamycin appears to be a very effective for controlling growth and ohcratoxin production by strains of *A. carbonarius.*	[2]
A glucose-yeast extract-salt medium	*Aspergillus parasiticus*	Natamycin (Pimaricin), with a low pH, low temperature or 4% or 6% NaCl, could initially slow mycelial growth and inhibit toxin production; the mold overcame the inhibitory effects.	[3]
Olive paste	*Aspergillus ochraceus*	Natamycin delayed growth and sporulation by *A. ochraceus*. Production of penicillic acid was also decreased.	[4]
Antifungal synergism:			
Apple juice	*Candida tropicalis, Candida krusei, Candida kefyr, Rhodotorula mucilaginosa*	The farnesol (yeast quorum sensing molecule) enhanced natamycin efficacy; reduced biofilm development from juice on stainless steel surfaces.	[5]
Blackberry fruits	*Aspergillus japonicus, Gilbertella persicaria*	Synergistic antifungal activity of ferulic acid and natamycin.	[6]
Phyllo pastry	Mesophilic total viable counts, yeasts and molds, psychrotrophic bacteria, lactic acid bacteria, Enterobacteriaceae, enterococci	The combination of chitosan and natamycin can delay the spoilage of phyllo pastry.	[7]
Galotyri cheese	Yeasts, molds	Natamycin, added alone or in combination with nisin, suppressed fungal growth.	[8]
Shiitake mushroom (*Lentinus edodes*)	Yeasts, molds	Mushroom coated with gum arabic + natamycin maintained tissue firmness and showed reduction in fungal counts.	[9]
Concord and Niagara grape juices	Yeast cocktail of *Dekkera*, *Kluveromyces*, *Brettanomyces*, and *Zygosaccharomyces*	Dimethyldicarbonate and natamycin combination treatments in still Niagara juice and in carbonated Concord and Niagara juices exhibited promising results.	[10]
Button mushroom (*Agaricus bisporus*)	Yeasts, molds	Treatment with natamycin + pure oxygen maintained tissue firmness, inhibited increase of respiration rate, delayed browning and cap opening, and reduced yeasts and molds.	[11]
Aqueous solutions	N/A	The natamycin:beta-cyclodextrin complex and natamycin:gamma-cyclodextrin complex	[12]

		were significantly more stable ($p < 0.05$) than natamycin in its free state; aqueous solutions stored in darkness at 4 ^{o}C.	
Formulated blend	N/A	Stability study of a nisin/natamycin blend; after 1 year, 50% of nisin and 90% of natamycin remained in the formulation. pH 5-6.	[13]
Natamycin sensitivity to low pHs & light:			
Agriculture and food. foster: Review	*Candida* spp.	Acidic pH natamycin degradation. The use of any anti-infective agent as a food preservative to be limited to an absolute minimum.	[14]
Wine	N/A	Detection method optimization; Instability in wine; light sensitivity.	[15]
Cow milk	N/A	Photoprotective effect of mycosporine-like amino acids for natamycin stability; protect cow milk exposed to light in refrigerated glass containers.	[16]
Effect of ingredients on natamycin efficacy:			
Simulated acid sauces	*Zygosaccharomyces bailii*	Efficacy of natamycin as a function of xanthan gum and sodium chloride concentrations.	[17]
Cheese-mimicking matrix	*Galactomyces geotrichum, Mucor racemosus, Penicillium commune, Phoma pinodella, Candida parapsilosis, Meyerozyma guilliermondii, Rhodotorula mucilaginosa, Trichosporon asahii, Yarrowia lipolytica*	The effect of the screening medium on natamycin minimum inhibitory concentration.	[18]
Table olive	*Saccharomyces cerevisiae, Wickerhamomyces anomalus, Candida boidinii*	Antagonistic effects between natamycin and citric acid in table olive packaging.	[19]
Induced tolerance to natamycin:			
Agar medium	*Saccharomyces cerevisiae, Candida parapsilosis, Candida albicans, Candida krusei, Rhodotorula mucilaginosa, Trichosporon asahii, Geotrichum candidum, Fusarium solani, Aspergillus terreus, Aspergillus fumigatus,*	Natamycin-exposure caused an increase of minimum inhibitory concentrations; a continuous and prolonged increasing selection pressure induced natamycin tolerance in fungi.	[20,21]

	Cladophialophora potulentorum, Neosartorya spinosa, Penicillium discolor, Mucor plumbeus, Aspergillus ochraceus, Verticillium fungicola, Colletotrichum musae, Fusarium oxysporum, Trichoderma aggressivum		
Natamycin-containing films/coatings:			
Cherry tomato	*Botrytis cinerea*	Natamycin/methyl-β-cyclodextrin coatings; inhibit fruit decay in tomato fruit during storage.	[22]
Cheese	*Penicillium* spp.	Corn starch-based coating with 0.1 % natamycin; mold control on semi-hard cheese during ripening.	[23]
Agar medium	*Aspergillus niger*	Natamycin-containing thermoplastic films; heat-pressed films had higher inhibition zone diameters than blown films.	[24]
Kashar cheese	Molds	Casein/natamycin edible films for mold control.	[25]
Commercial semi-soft cheese; food packaging	Molds	Natamycin based sol-gel antimicrobial coatings on polylactic acid films.	[26]
Tapioca starch edible films	N/A	Effect of natamycin on the physicochemical properties of edible films.	[27]
Gliadin films	*Penicillium* sp., *Alternaria solani, Colletotrichum acutatum*	Incorporation of natamycin to films resulted in greater water uptake, weight loss/diameter gain, higher water vapor and oxygen permeabilities; a looser packing of the protein chains by natamycin.	[28]
Cheeses	Yeasts, molds	Antimicrobial edible coatings inhibited the growth of pathogenic or contaminant microorganisms (yeasts and molds).	[29]
Natamycin modes of action in foods:			
Malt extract	*Penicillium discolor*	Unlike other polyene drugs such as nystatin and filipin, natamycin cannot permeabilize germinating conidia, but disrupts endocytosis.	[30]
Commercially manufactured poultry feed	*Aspergillus fumigatus, Aspergillus parasiticus, Aspergillus flavus, Paecilomyces spp., Rhizopus spp., Fusarium moniliforme, Penicillium rubrum*	The growth inhibition by natamycin was more pronounced compared with the sporicidal activity.	[31]
Mandarin fruit	*Botrytis cinerea*	Postharvest disease control; management of pathogens resistant to fungicides with different modes of action.	[32]
Others:			

Morus notabilis Schneid. Daye	Fungal mixture	Mulberry fruit postharvest decay control.	[**33**]
Strawberry crown Rot	QoI-Resistant *Colletotrichum acutatum*	Disease severity and plant mortality in field studies were reduced;<br>Fruit yield was significantly increased.	[34]
Livestock food systems: Review	*Aspergillus flavus, Aspergillus paraciticus*	Natamycin (and nisin): the only natural preservatives being regulated.	[35]
Food-producing industry	*Candida* spp., *Aspergillus fumigatus*	Natamycin has to be used cautiously by the food industry and that exposure of the human resident flora should be minimized extensively in order to maintain the life-saving potential of polyenes.	[36,37]
Yogurt	N/A	Natamycin content and quality of yogurt.	[38]
Artificial diet	*Aspergillus niger*	Natamycin for the rearing of rice leaf folder.	[39]
Greek traditional deli salad "Tzatziki"	Yeasts, molds	The shelf-life of Tzatziki was extended by ca. 5-6 days (natamycin, citrus and citrus plus natamycin).	[40]
Iranian Yogurt Drink	N/A	Natamaycin was detected in 10.25% of the samples.	[41]
South African fruit juices	N/A	Detection of natamycin above the legal limits in some samples.	[42]
Natural black olive fermentation	Yeasts, molds	Natamycin inhibited the growth of fungi.	[43]
Orange juice	*Pichia fermentans*	The effect of natamycin-pulsed electric fields (PEF) combination against *P. fermentans* was not significantly different to that of PEF alone.	[44]
Soil of a vegetable field	*Fusarium oxysporum, Botrytis cinerea, Monilinia laxa*	Application as a biological control agent for fungal plant diseases.	[45]
Shredded Cheddar cheese	Molds	The semisynthetic natamycin derivative is less effective than the parent natamycin.	[46]
Serum dextrose agar With *Brucella* selective supplement	*Brucella abortus, Brucella melitensis*	Natamycin would be an alternative to cycloheximide.	[47]
Argentinian cheese factory	*Phoma glomerata*	0.5% (w/v) Natamycin was effective to treat *P. glomerata.*	[48]
Agar medium	*Aspergillus flavus, Aspergillus parasiticus, Aspergillus ochraceus, Penicillium* sp. M46, *Penicillium patulum, Penicillium roquefortii, Penicillium citrinum*	Antimycotic effect of natamycin.	[49,50]
Italian dry sausage	Molds	Antimycotic activity.	[51]
Cheese Warehouses	*Acremomium, sclerotigenum, Aspergillus versicolor, Beauverla alba, Cladosporium cladosporioides, Penicillium brevicompactum,*	Antimycotic activity.	[52]

	Penicillium cf. *lividum, Penicillium chrysogenum, Penicillium nigricans, Penicillium citreoviride, Penicillium roseopurpureum, Penicillium verrucosum, Penicillium viridicatum, Scopulariopsis asperula*		
Cottage cheese	*Aspergillus niger, Saccharomyces cerevisiae*	Antimycotic activity.	[53]
Natamycin detection methods in foods:			
Dairy products (Cheese, yogurt)	N/A	Determination of preservatives in dairy Products by HPLC and Chemometric analysis.	[54]
Cheeses	N/A	A liquid chromatography-tandem mass spectrometric with electrospray ionization method for detecting preservatives (benzoic acid, citric acid, hexamethylenetetramine, lysozyme, natamycin, nisin and sorbic acid) in cheese.	[55]
Wines	N/A	A novel method for determination of natamycin in wines; ultrahigh-performance liquid chromatography coupled to tandem mass spectrometry.	[56]
Dulce de leche, a traditional South American product	N/A	Liquid chromatography-tandem mass spectrometry method for the simultaneous determination of sorbic acid, natamycin and tylosin.	[57]
Hard and pasta filata cheeses	N/A	RP-HPLC with UV detection of natamycin.	[58]
Cheese, cheese rind	N/A	A rapid spectrophotometric method for determining natamycin. Quantitation and detection-limits were estimated at 0.5 and 0.25 mg/kg, respectively.	[59]
Cheese, cheese rind	N/A	Spectrometric method gave good results; liquid chromatographic method with ultraviolet detection gave reasonable results.	[60,61]
Cheese	N/A	A high-performance liquid chromatographic method for natamycin detection.	[62]

Table S2. Examples of differential antifungal efficacy of drugs/preservatives affected by different pHs.

Drug/compound tested	Drug/compound characteristics	Fungi	Low pH	High pH	Mechanisms	References
5-Flucytosine (5-FC)	Derivative of the nucleobase cytosine	*Aspergillus fumigatus*	High efficacy of 5-FC at pH 5	Low efficacy of 5-FC at pH 7	Down regulation of *fcyB* gene encoding a purine-cytosine permease (orthologous to 5-FC importers) at pH 7	[63]
Naftifine	Allylamine derivative	*Trichophyton mentagrophytes* var. *quinckeanum*, *Microsporum racemosum*	Low efficacy at pH 4	High efficacy at pH 8	-	[64]
Methotrexate (MTX)	Antifolate	*Candida albicans*, *Candida tropicalis*, *Candida parapsilosis*	High efficacy at pH 5	Low efficacy at pH 7	The pH-dependent antifungal activity of MTX is via differential cellular uptake or permeability and is species specific. *Candida glabrata* was insensitive at either pH.	[65]
Valproic acid (VPA)	Antipsychotic drug	*C. albicans*	High efficacy at acidic pH (pH 4.5)	Low efficacy at pH 8.0	VPA alters vacuole integrity. VPA synergizes with the terbinafine (allylamine antifungal).	[66]
Dimethylaminododecyl methacrylate (DMADDM),	Added to acrylic resin (oral) as a non-releasing antifungal agent	*C. albicans* (planktonic and biofilm)	Low efficacy at pH 4	High efficacy at alkaline pH (pH 7 to 9)	The pH values and pH-regulated genes affect the zeta potential of fungal cells, thus the binding between DMADDM and cells.	[67]
Potassium sorbate	Food preservative	*Aspergillus flavus*	High efficacy at pH 3.0	Low efficacy at pH 4.5	-	[68]
Sodium sulfite, Sodium propionate	Food preservatives	*A. flavus*, *Aspergillus parasiticus*	High efficacy at pH 3.0	Low efficacy at pH 5.0 to 7.0	-	[69]

Chloroquine (CQ)	Antimalarial drug	*A. fumigatus, Aspergillus nidulans*	No significant difference in antifungal efficacy against *A. fumigatus* versus *A. nidulans* at pH 6	*A. fumigatus* is more susceptible to CQ than *A. nidulans* at pH 8	A higher extracellular pH enabled increased diffusion rate of CQ into the fungal organisms.	[70]

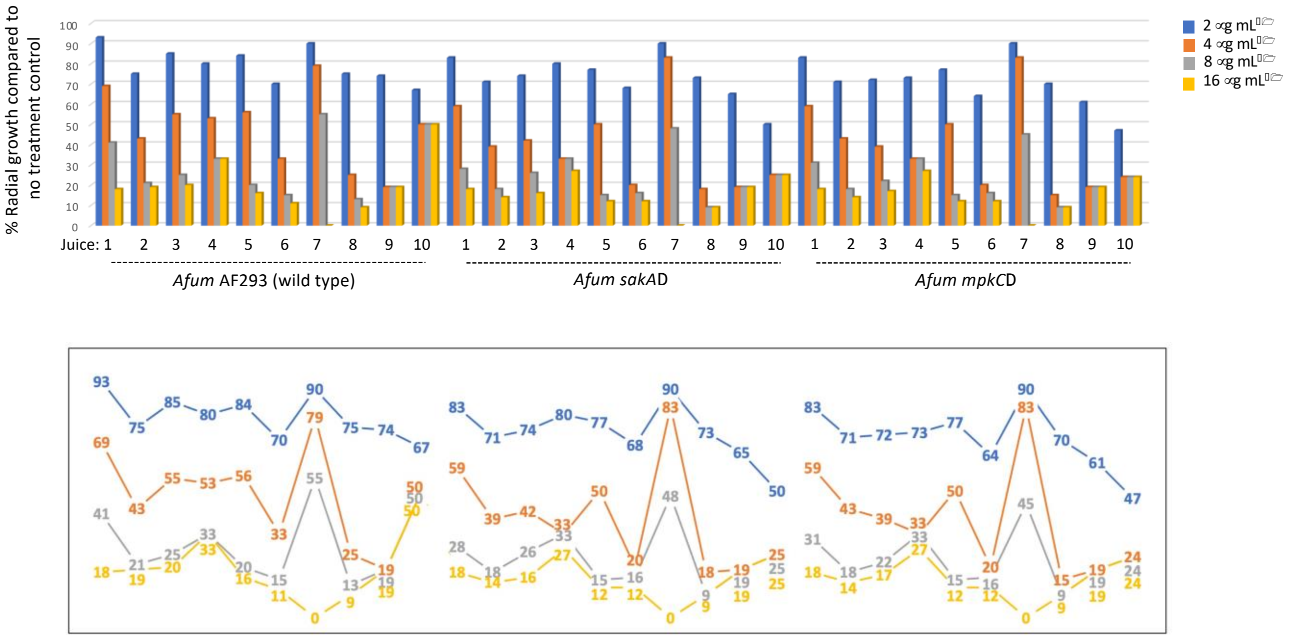

Figure S1. Susceptibility of *A. fumigatus* AF293 (wild type) and MAPK mutants (*sakA* , *mpkC*) to natamycin (NAT) (2, 4, 8, 16 g mL^{-1}) tested in the commercial, organic fruit juices (triplicate) (SD < 2%). Lower panel: Line graph showing numerical values of % radial growth at each test condition.

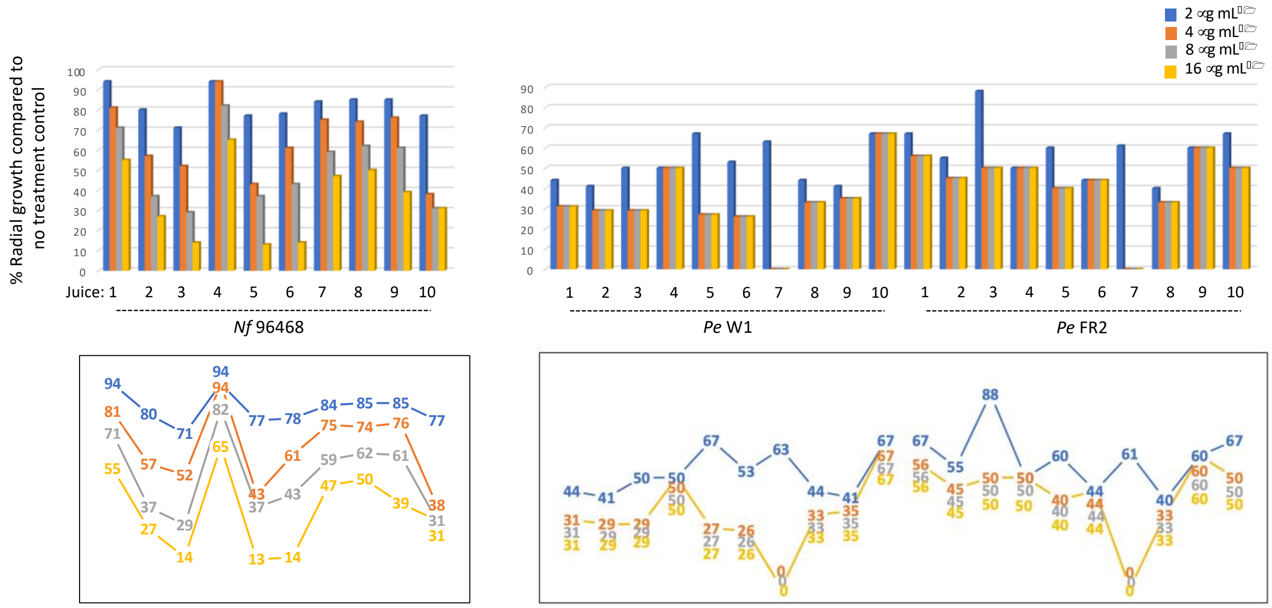

Figure S2. Susceptibility of foodborne fungal contaminants (*Pe, P. expansum*; *Nf, N. fischeri*) to natamycin (NAT) (2, 4, 8, 16 g mL^{-1}) tested in the commercial, organic fruit juices (triplicate) (SD < 2%). Lower panel: Line graph showing numerical values of % radial growth at each test condition.

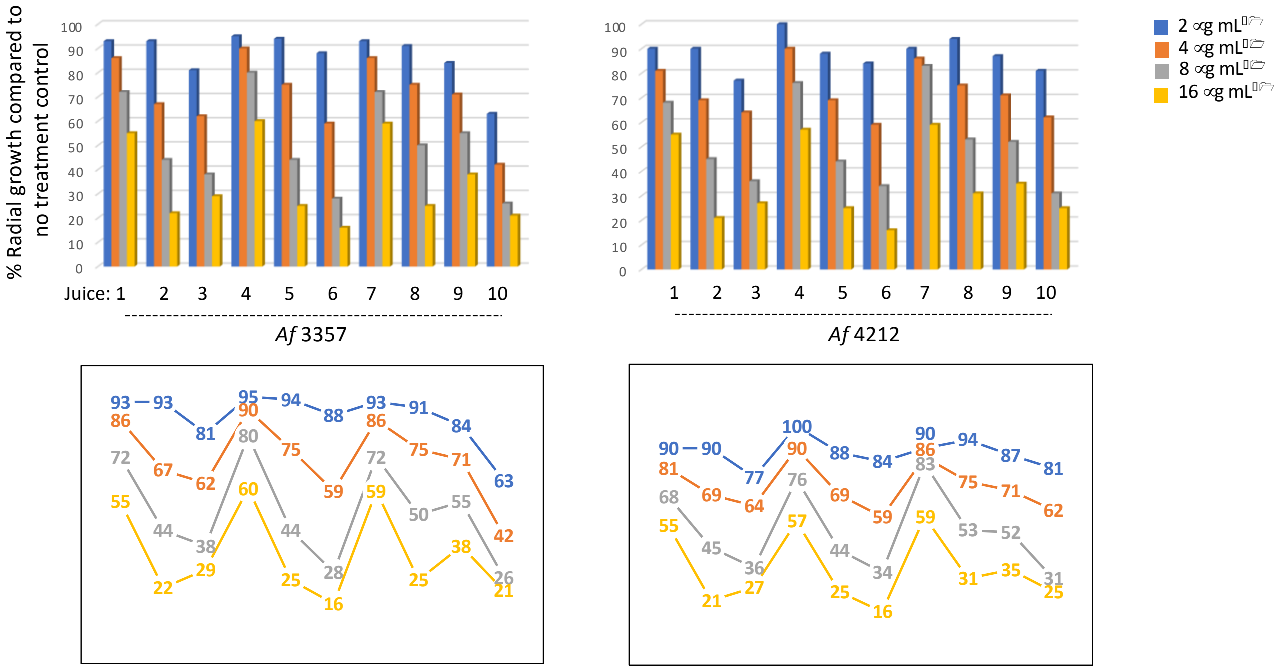

Figure S3. Susceptibility of foodborne fungal contaminants (*A. flavus* NRRL 3357, NRRL 4212) to natamycin (NAT) (2, 4, 8, 16 g mL^{-1}) tested in the commercial, organic fruit juices (triplicate) (SD < 2%). Lower panel: Line graph showing numerical values of % radial growth at each test condition.

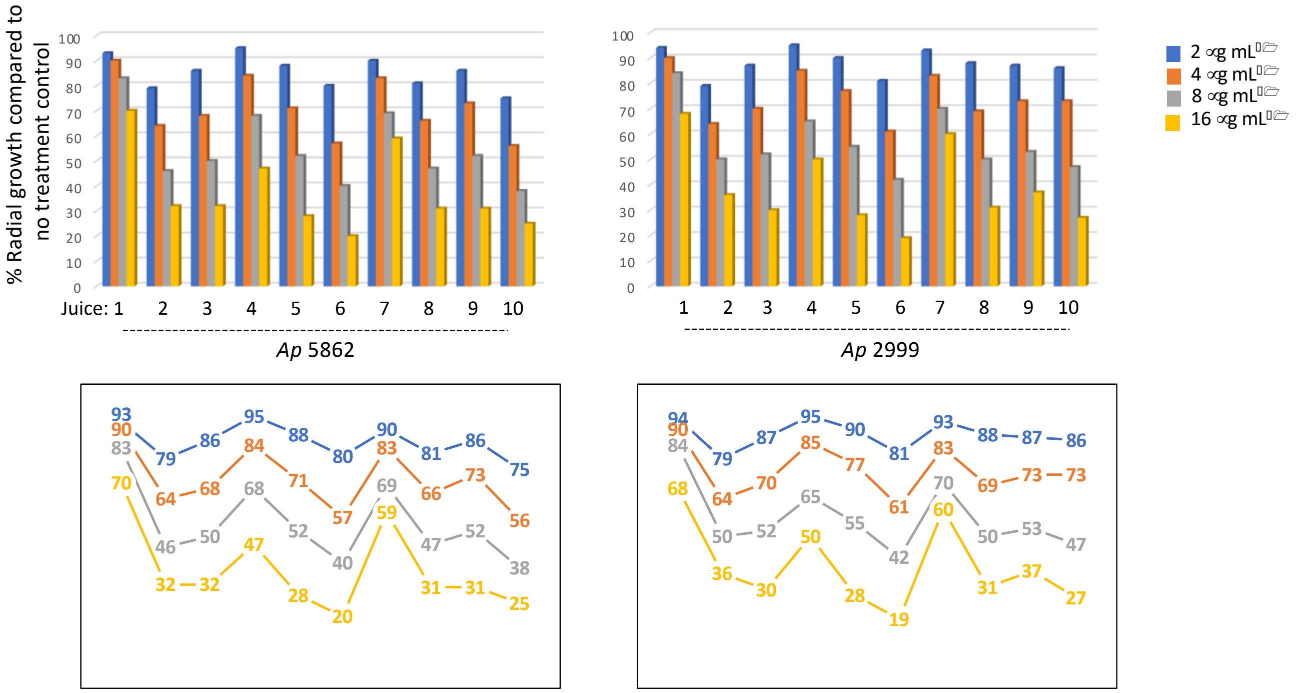

Figure S4. Susceptibility of foodborne fungal contaminants (*A. parasiticus* NRRL 2999, NRRL 5862) to natamycin (NAT) (2, 4, 8, 16 g mL^{-1}) tested in the commercial, organic fruit juices (triplicate) (SD < 2%). Lower panel: Line graph showing numerical values of % radial growth at each test condition.

References

1. Mahjoub, A.; Bullerman, L.B. Effects of natamycin and potassium sorbate on growth and aflatoxin production in olives. *Arch. Inst. Pasteur Tunis* **1986**, *63*, 513–525.
2. Medina, A.; Jiménez, M.; Mateo, R.; Magan, N. Efficacy of natamycin for control of growth and ochratoxin A production by *Aspergillus carbonarius* strains under different environmental conditions. *J. Appl. Microbiol.* **2007**, *103*, 2234–2239, doi:10.1111/j.1365-2672.2007.03462.x.
3. Rusul, G.; Marth, E.H. Growth and aflatoxin production by *Aspergillus parasiticus* in a medium at different pH values and with or without pimaricin. *Z. Lebensm. Unters. Forsch.* **1988**, *187*, 436–439, doi:10.1007/bf01122645.
4. Gourama, H.; Bullerman, L.B. Effects of Potassium Sorbate and Natamycin on Growth and Penicillic Acid Production by *Aspergillus ochraceus* (1). *J. Food Prot.* **1988**, *51*, 139–144, doi:10.4315/0362-028x-51.2.139.
5. Agustín, M.D.R.; Viceconte, F.R.; Vela Gurovic, M.S.; Costantino, A.; Brugnoni, L.I. Effect of quorum sensing molecules and natamycin on biofilms of *Candida tropicalis* and other yeasts isolated from industrial juice filtration membranes. *J. Appl. Microbiol.* **2019**, *126*, 1808–1820, doi:10.1111/jam.14248.
6. Liu, H.; Zhao, H.; Lyu, L.; Huang, Z.; Fan, S.; Wu, W.; Li, W. Synergistic effect of natural antifungal agents for postharvest diseases of blackberry fruits. *J. Sci. Food Agric.* **2019**, *99*, 3343–3349, doi:10.1002/jsfa.9551.
7. Tsiraki, M.I.; El-Obeid, T.; Yehia, H.M.; Karam, L.; Savvaidis, I.N. Effects of Chitosan and Natamycin on Vacuum-Packaged Phyllo: A Pastry Product. *J. Food Prot.* **2018**, *81*, 1982–1987, doi:10.4315/0362-028x.Jfp-18-236.
8. Kallinteri, L.D.; Kostoula, O.K.; Savvaidis, I.N. Efficacy of nisin and/or natamycin to improve the shelf-life of Galotyri cheese. *Food Microbiol.* **2013**, *36*, 176–181, doi:10.1016/j.fm.2013.05.006.
9. Jiang, T.; Feng, L.; Zheng, X.; Li, J. Physicochemical responses and microbial characteristics of shiitake mushroom (*Lentinus edodes*) to gum arabic coating enriched with natamycin during storage. *Food Chem.* **2013**, *138*, 1992–1997, doi:10.1016/j.foodchem.2012.11.043.
10. Siricururatana, P.; Iyer, M.M.; Manns, D.C.; Churey, J.J.; Worobo, R.W.; Padilla-Zakour, O.I. Shelf-life evaluation of natural antimicrobials for Concord and Niagara grape juices. *J. Food Prot.* **2013**, *76*, 72–78, doi:10.4315/0362-028x.Jfp-12-144.
11. Jiang, T. Effect of natamycin in combination with pure oxygen treatment on postharvest quality and selected enzyme activities of button mushroom (*Agaricus bisporus*). *J. Agric. Food Chem.* **2012**, *60*, 2562–2568, doi:10.1021/jf205160c.
12. Koontz, J.L.; Marcy, J.E.; Barbeau, W.E.; Duncan, S.E. Stability of natamycin and its cyclodextrin inclusion complexes in aqueous solution. *J. Agric. Food Chem.* **2003**, *51*, 7111–7114, doi:10.1021/jf030333q.
13. Duchateau, A.L.L.; van Scheppingen, W.B. Stability study of a nisin/natamycin blend by LC-MS. *Food Chem.* **2018**, *266*, 240–244, doi:10.1016/j.foodchem.2018.05.121.
14. Dalhoff, A. Does the use of antifungal agents in agriculture and food foster polyene resistance development? A reason for concern. *J. Glob. Antimicrob. Resist.* **2018**, *13*, 40–48, doi:10.1016/j.jgar.2017.10.024.
15. Sun, X.; Li, X.; Wang, P.; Ma, T.; Huang, W.; Han, S.; Zhan, J. Detection method optimization, content analysis and stability exploration of natamycin in wine. *Food Chem.* **2016**, *194*, 928–937, doi:10.1016/j.foodchem.2015.08.116.
16. Teixeira, G.H.A.; O'Keefe, S.F. Short communication: Mycosporine-like amino acids protect natamycin against photodegradation in milk exposed to fluorescent or light-emitting diode light. *J. Dairy Sci.* **2019**, *102*, 4972–4977, doi:10.3168/jds.2018-15658.
17. Zalazar, A.L.; Gliemmo, M.F.; Soria, M.; Campos, C.A. Modelling growth/no growth interface of *Zygosaccharomyces bailii* in simulated acid sauces as a function of natamycin, xanthan gum and sodium chloride concentrations. *Food Res. Int.* **2019**, *116*, 916–924, doi:10.1016/j.foodres.2018.09.028.
18. Garnier, L.; Salas, M.L.; Pinon, N.; Wiernasz, N.; Pawtowski, A.; Coton, E.; Mounier, J.; Valence, F. Technical note: High-throughput method for antifungal activity screening in a cheese-mimicking model. *J. Dairy Sci.* **2018**, *101*, 4971–4976, doi:10.3168/jds.2017-13518.
19. Arroyo-López, F.N.; Bautista-Gallego, J.; Romero-Gil, V.; Rodríguez-Gómez, F.; Garrido-Fernández, A. Growth/no growth interfaces of table olive related yeasts for natamycin, citric acid and sodium chloride. *Int. J. Food Microbiol.* **2012**, *155*, 257–262, doi:10.1016/j.ijfoodmicro.2012.02.007.
20. Streekstra, H.; Keuter, A.; Wilms, L. Reaction to Dalhoff and Levy: 'Does use of the polyene natamycin as a food preservative jeopardise the clinical efficacy of amphotericin B? A word of concern'. *Int. J. Antimicrob. Agents* **2015**, *46*, 595–596, doi:10.1016/j.ijantimicag.2015.07.005.
21. Streekstra, H.; Verkennis, A.E.; Jacobs, R.; Dekker, A.; Stark, J.; Dijksterhuis, J. Fungal strains and the development of tolerance against natamycin. *Int. J. Food Microbiol.* **2016**, *238*, 15–22, doi:10.1016/j.ijfoodmicro.2016.08.006.

22. Yang, Y.; Huan, C.; Liang, X.; Fang, S.; Wang, J.; Chen, J. Development of Starch-Based Antifungal Coatings by Incorporation of Natamycin/Methyl-β-Cyclodextrin Inclusion Complex for Postharvest Treatments on Cherry Tomato against *Botrytis cinerea*. *Molecules* **2019**, *24*, 3962, doi:10.3390/molecules24213962.
23. González-Forte, L.D.S.; Amalvy, J.I.; Bertola, N. Corn starch-based coating enriched with natamycin as an active compound to control mold contamination on semi-hard cheese during ripening. *Heliyon* **2019**, *5*, e01957, doi:10.1016/j.heliyon.2019.e01957.
24. Colak, B.Y.; Peynichou, P.; Galland, S.; Oulahal, N.; Prochazka, F.; Degraeve, P. Antimicrobial Activity of Nisin and Natamycin Incorporated Sodium Caseinate Extrusion-Blown Films: A Comparative Study with Heat-Pressed/Solution Cast Films. *J. Food Sci.* **2016**, *81*, E1141–E1150, doi:10.1111/1750-3841.13284.
25. Yangılar, F.; Oğuzhan Yıldız, P. Casein/natamycin edible films efficiency for controlling mould growth and on microbiological, chemical and sensory properties during the ripening of Kashar cheese. *J. Sci. Food Agric.* **2016**, *96*, 2328–2336, doi:10.1002/jsfa.7348.
26. Lantano, C.; Alfieri, I.; Cavazza, A.; Corradini, C.; Lorenzi, A.; Zucchetto, N.; Montenero, A. Natamycin based sol-gel antimicrobial coatings on polylactic acid films for food packaging. *Food Chem.* **2014**, *165*, 342–347, doi:10.1016/j.foodchem.2014.05.066.
27. Ollé Resa, C.P.; Jagus, R.J.; Gerschenson, L.N. Effect of natamycin, nisin and glycerol on the physicochemical properties, roughness and hydrophobicity of tapioca starch edible films. *Mater Sci. Eng. C Mater Biol. Appl.* **2014**, *40*, 281–287, doi:10.1016/j.msec.2014.04.005.
28. Balaguer, M.P.; Fajardo, P.; Gartner, H.; Gomez-Estaca, J.; Gavara, R.; Almenar, E.; Hernandez-Munoz, P. Functional properties and antifungal activity of films based on gliadins containing cinnamaldehyde and natamycin. *Int. J. Food Microbiol.* **2014**, *173*, 62–71, doi:10.1016/j.ijfoodmicro.2013.12.013.
29. Ramos, Ó.; L.; Pereira, J.O.; Silva, S.I.; Fernandes, J.C.; Franco, M.I.; Lopes-da-Silva, J.A.; Pintado, M.E.; Malcata, F.X. Evaluation of antimicrobial edible coatings from a whey protein isolate base to improve the shelf life of cheese. *J. Dairy Sci.* **2012**, *95*, 6282–6292, doi:10.3168/jds.2012-5478.
30. Van Leeuwen, M.R.; Golovina, E.A.; Dijksterhuis, J. The polyene antimycotics nystatin and filipin disrupt the plasma membrane, whereas natamycin inhibits endocytosis in germinating conidia of *Penicillium discolor*. *J. Appl. Microbiol.* **2009**, *106*, 1908–1918, doi:10.1111/j.1365-2672.2009.04165.x.
31. Brothers, A.M.; Wyatt, R.D. The antifungal activity of natamycin toward molds isolated from commercially manufactured poultry feed. *Avian Dis.* **2000**, *44*, 490–497, doi:10.2307/1593087.
32. Saito, S.; Wang, F.; Xiao, C.L. Efficacy of Natamycin Against Gray Mold of Stored Mandarin Fruit Caused by Isolates of *Botrytis cinerea* With Multiple Fungicide Resistance. *Plant Dis.* **2020**, *104*, 787–792, doi:10.1094/pdis-04-19-0844-re.
33. Wen, M.; Lin, X.; Yu, Y.; Wu, J.; Xu, Y.; Xiao, G. Natamycin treatment reduces the quality changes of postharvest mulberry fruit during storage. *J. Food Biochem.* **2019**, *43*, e12934, doi:10.1111/jfbc.12934.
34. Haack, S.E.; Ivors, K.L.; Holmes, G.J.; Förster, H.; Adaskaveg, J.E. Natamycin, a New Biofungicide for Managing Crown Rot of Strawberry Caused by QoI-Resistant Colletotrichum acutatum. Plant Dis. 2018, 102, 1687–1695, doi:10.1094/pdis-12-17-2033-re..
35. Lee, N.K.; Paik, H.D. Status, Antimicrobial Mechanism, and Regulation of Natural Preservatives in Livestock Food Systems. *Korean J. Food Sci. Anim. Resour.* **2016**, *36*, 547–557, doi:10.5851/kosfa.2016.36.4.547.
36. Dalhoff, A. Response to the reaction to Dalhoff and Levy: 'Does use of the polyene natamycin as a food preservative jeopardise the clinical efficacy of amphotericin B? A word of concern'. *Int. J. Antimicrob. Agents* **2015**, *46*, 596–597, https://doi.org/10.1016/j.ijantimicag.2015.07.006.
37. Dalhoff, A.A.; Levy, S.B. Does use of the polyene natamycin as a food preservative jeopardise the clinical efficacy of amphotericin B? A word of concern. *Int. J. Antimicrob. Agents* **2015**, *45*, 564–567, doi:10.1016/j.ijantimicag.2015.02.011.
38. Dervisoglu, M.; Gul, O.; Aydemir, O.; Yazici, F.; Kahyaoglu, T. Natamycin content and quality evaluation of yoghurt from small- and large-scale brands in Turkey. *Food Addit. Contam. Part B Surveill.* **2014**, *7*, 254–260, doi:10.1080/19393210.2014.901426.
39. Su, J.; Wang, Y.C.; Zhang, S.K.; Ren, X.B. Antifungal agents against *Aspergillus niger* for rearing rice leaffolder larvae (Lepidoptera: Pyralidae) on artificial diet. *J. Econ. Entomol.* **2014**, *107*, 1092–1100, doi:10.1603/ec13296.
40. Tsiraki, M.I.; Savvaidis, I.N. Citrus extract or natamycin treatments on "Tzatziki"—A traditional Greek salad. *Food Chem.* **2014**, *142*, 416–422, doi:10.1016/j.foodchem.2013.07.087.
41. Esfandiari, Z.; Badiey, M.; Mahmoodian, P.; Sarhangpour, R.; Yazdani, E.; Mirlohi, M. Simultaneous Determination of Sodium Benzoate, Potassium Sorbate and Natamycin Content in Iranian Yoghurt Drink (Doogh) and the Associated Risk of Their Intake through Doogh Consumption. *Iran J. Public Health* **2013**, *42*, 915–920.

42. Stander, M.A.; Kühn, W.; Hiten, N.F. Survey of South African fruit juices using a fast screening HILIC-MS method. *Food Addit. Contam. Part A Chem. Anal. Control Expo. Risk Assess.* **2013**, *30*, 1473–1484, doi:10.1080/19440049.2013.811545.
43. Hondrodimou, O.; Kourkoutas, Y.; Panagou, E.Z. Efficacy of natamycin to control fungal growth in natural black olive fermentation. *Food Microbiol.* **2011**, *28*, 621–627, doi:10.1016/j.fm.2010.11.015.
44. McNamee, C.; Noci, F.; Cronin, D.A.; Lyng, J.G.; Morgan, D.J.; Scannell, A.G. PEF based hurdle strategy to control *Pichia fermentans*, *Listeria innocua* and *Escherichia coli* k12 in orange juice. *Int. J. Food Microbiol.* **2010**, *138*, 13–18, doi:10.1016/j.ijfoodmicro.2009.12.001.
45. Lu, C.G.; Liu, W.C.; Qiu, J.Y.; Wang, H.M.; Liu, T.; De Liu, W. Identification of an antifungal metabolite produced by a potential biocontrol Actinomyces strain A01. *Braz. J. Microbiol.* **2008**, *39*, 701–707, doi:10.1590/s1517-838220080004000020.
46. Suloff, E.C.; Marcy, J.E.; Hackney, C.R.; Sumner, S.S.; Bishop, J.R. Comparative study of a semisynthetic derivative of natamycin and the parent antibiotic on the spoilage of shredded cheddar cheese. *J. Food Prot.* **2003**, *66*, 1499–1502, doi:10.4315/0362-028x-66.8.1499.
47. Stack, J.A.; Harrison, M.; Perrett, L.L. Evaluation of a selective medium for Brucella isolation using natamycin. *J. Appl. Microbiol.* **2002**, *92*, 724–728, doi:10.1046/j.1365-2672.2002.01595.x.
48. Basílico, J.C.; DeBasílico, M.Z.; Chiericatti, C.; Vinderola, C.G. Characterization and control of thread mould in cheese. *Lett. Appl. Microbiol.* **2001**, *32*, 419–423, doi:10.1046/j.1472-765x.2001.00934.x.
49. Azzouz, M.A.; Bullerman, L.B. Comparative Antimycotic Effects of Selected Herbs, Spices, Plant Components and Commercial Antifungal Agents (1). *J. Food Prot.* **1982**, *45*, 1298–1301, doi:10.4315/0362-028x-45.14.1298.
50. Ray, L.L.; Bullerman, L.B. Preventing Growth of Potentially Toxic Molds Using Antifungal Agents (1). *J. Food Prot.* **1982**, *45*, 953–963, doi:10.4315/0362-028x-45.10.953.
51. Holley, R.A. Prevention of surface mold growth on Italian dry sausage by natamycin and potassium sorbate. *Appl. Environ. Microbiol.* **1981**, *41*, 422–429, doi:10.1128/aem.41.2.422-429.1981.
52. de Boer, E.; Stolk-Horsthuis, M. Sensitivity to Natamycin[1] (Pimaricin) of Fungi Isolated in Cheese Warehouses. *J. Food Prot.* **1977**, *40*, 533–536, doi:10.4315/0362-028x-40.8.533.
53. Nilson, K.M.; Shahani, K.M.; Vakil, J.R.; Kilara, A. Pimaricin and mycostatin for retarding cottage cheese spoilage. *J. Dairy Sci.* **1975**, *58*, 668–671, doi:10.3168/jds.S0022-0302(75)84625-X.
54. Zamani Mazdeh, F.; Sasanfar, S.; Chalipour, A.; Pirhadi, E.; Yahyapour, G.; Mohammadi, A.; Rostami, A.; Amini, M.; Hajimahmoodi, M. Simultaneous Determination of Preservatives in Dairy Products by HPLC and Chemometric Analysis. *Int. J .Anal. Chem.* **2017**, *2017*, 3084359, doi:10.1155/2017/3084359.
55. Fuselli, F.; Guarino, C.; La Mantia, A.; Longo, L.; Faberi, A.; Marianella, R.M. Multi-detection of preservatives in cheeses by liquid chromatography-tandem mass spectrometry. *J. Chromatogr. B Analyt. Technol. Biomed. Life Sci.* **2012**, *906*, 9–18, doi:10.1016/j.jchromb.2012.07.035.
56. Repizo, L.M.; Martinez, L.D.; Olsina, R.A.; Cerutti, S.; Raba, J. A novel and rapid method for determination of natamycin in wines based on ultrahigh-performance liquid chromatography coupled to tandem mass spectrometry: Validation according to the 2002/657/EC European decision. *Anal. Bioanal. Chem.* **2012**, *402*, 965–973, doi:10.1007/s00216-011-5481-6.
57. Molognoni, L.; Valese, A.C.; Lorenzetti, A.; Daguer, H.; De Dea Lindner, J. Development of a LC-MS/MS method for the simultaneous determination of sorbic acid, natamycin and tylosin in Dulce de leche. *Food Chem.* **2016**, *211*, 748–756, doi:10.1016/j.foodchem.2016.05.105.
58. Guarino, C.; Fuselli, F.; Mantia, A.L.; Longo, L. Development of an RP-HPLC method for the simultaneous determination of benzoic acid, sorbic acid, natamycin and lysozyme in hard and pasta filata cheeses. *Food Chem.* **2011**, *127*, 1294–1299, doi:10.1016/j.foodchem.2011.01.086.
59. Fletouris, D.J.; Botsoglou, N.A.; Mantis, A.J. Rapid spectrophotometric method for analyzing natamycin in cheese and cheese rind. *J. AOAC Int.* **1995**, *78*, 1024–1029.
60. de Ruig, W.G. Determination of natamycin in cheese and cheese rind: Interlaboratory collaborative study. *J. Assoc. Off. Anal. Chem.* **1987**, *70*, 949–954.
61. De Ruig, W.G.; van Oostrom, J.J.; Leenheer, K. Spectrometric and liquid chromatographic determination of natamycin in cheese and cheese rind. *J. Assoc. Off. Anal. Chem.* **1987**, *70*, 944–948.
62. Tuinstra, L.G.; Traag, W.A. Liquid chromatographic determination of natamycin in cheese at residue levels. *J. Assoc. Off. Anal. Chem.* **1982**, *65*, 820–822.
63. Gsaller, F.; Furukawa, T.; Carr, P.D.; Rash, B.; Jöchl, C.; Bertuzzi, M.; Bignell, E.M.; Bromley, M.J. Mechanistic Basis of pH-Dependent 5-Flucytosine Resistance in *Aspergillus fumigatus*. *Antimicrob. Agents Chemother.* **2018**, *62*, e02593-02517, doi:10.1128/aac.02593-17.

64. Georgopoulos, A.; Petranyi, G.; Mieth, H.; Drews, J. In vitro activity of naftifine, a new antifungal agent. *Antimicrob. Agents Chemother.* **1981**, *19*, 386–389, doi:10.1128/aac.19.3.386.
65. DeJarnette, C.; Luna-Tapia, A.; Estredge, L.R.; Palmer, G.E. Dihydrofolate Reductase Is a Valid Target for Antifungal Development in the Human Pathogen *Candida albicans*. *mSphere* **2020**, *5*, e00374-00320, doi:10.1128/mSphere.00374-20.
66. Chaillot, J.; Tebbji, F.; García, C.; Wurtele, H.; Pelletier, R.; Sellam, A. pH-Dependant Antifungal Activity of Valproic Acid against the Human Fungal Pathogen *Candida albicans*. *Front. Microbiol.* **2017**, *8*, doi:10.3389/fmicb.2017.01956.
67. Chen, H.; Zhou, Y.; Zhou, X.; Liao, B.; Xu, H.H.K.; Chu, C.-H.; Cheng, L.; Ren, B. Dimethylaminododecyl methacrylate inhibits *Candida albicans* and oropharyngeal candidiasis in a pH-dependent manner. *Appl. Microbiol. Biotechnol.* **2020**, *104*, 3585–3595, doi:10.1007/s00253-020-10496-0.
68. Nguefack, J.; Leth, V.; Amvam Zollo, P.H.; Mathur, S.B. Evaluation of five essential oils from aromatic plants of Cameroon for controlling food spoilage and mycotoxin producing fungi. *Int. J. Food Microbiol.* **2004**, *94*, 329–334, doi:10.1016/j.ijfoodmicro.2004.02.017.
69. Holmquist, G.U.; Walker, H.W.; Stahr, H.M. Influence of Temperature, pH, Water Activity and Antifungal Agents on Growth of *Aspergillus flavus* and *A. parasiticus*. *J. Food Sci.* **1983**, *48*, 778–782, doi:10.1111/j.1365-2621.1983.tb14897.x.
70. Henriet, S.S.V.; Jans, J.; Simonetti, E.; Kwon-Chung, K.J.; Rijs, A.J.M.M.; Hermans, P.W.M.; Holland, S.M.; de Jonge, M.I.; Warris, A. Chloroquine Modulates the Fungal Immune Response in Phagocytic Cells From Patients With Chronic Granulomatous Disease. *J. Infect. Dis.* **2013**, *207*, 1932–1939, doi:10.1093/infdis/jit103.
